# IL-33-Induced Cytokine Secretion and Survival of Mouse Eosinophils Is Promoted by Autocrine GM-CSF

**DOI:** 10.1371/journal.pone.0163751

**Published:** 2016-09-30

**Authors:** Ralf Willebrand, David Voehringer

**Affiliations:** Department of Infection Biology, University Hospital Erlangen, Friedrich-Alexander University Erlangen-Nuremberg (FAU), Erlangen, Germany; Centre National de la Recherche Scientifique, FRANCE

## Abstract

Eosinophils are major effector cells during allergic responses and helminth infections. Recent studies further highlight eosinophils as important players in many other biological processes. Therefore it is important to understand how these cells can be modulated in terms of survival and effector function. In the present study we investigated how eosinophils respond to the alarmin IL-33. We show that IL-33 promotes eosinophil survival in a ST2- and MyD88-dependent manner. IL-33-mediated protection from apoptosis was dependent on autocrine GM-CSF release. In addition, GM-CSF increased the IL-33-induced secretion of IL-4 and IL-13 from eosinophils. Unexpectedly, this effect was further enhanced by cross-linking of Siglec-F, a proposed inhibitory and apopotosis-inducing receptor on eosinophils. Co-culture experiments with eosinophils and macrophages revealed that the IL-33-induced release of IL-4 and IL-13 from eosinophils was required for differentiation of alternatively activated macrophages (AAMs). The differentiation of AAMs could be further increased in the presence of GM-CSF. These results indicate that cross-talk between Siglec-F and the receptors for IL-33, LPS and GM-CSF plays an important role for efficient secretion of IL-4 and IL-13. Deciphering the molecular details of this cross-talk could lead to the development of new therapeutic option to treat eosinophil-associated diseases.

## Introduction

The alarmin IL-33 is a member of the IL-1 family which is rapidly released during tissue damage and serves as an important mediator of barrier immunity in skin, lung and intestine [1]. IL-33 acts on several different cell types of the innate and adaptive immune system by binding to the IL-33 receptor which consists of the transmembrane form of the ST2 molecule in association with the IL-1 receptor accessory protein (IL-1RAcP) [2]. Downstream signaling is mediated in part by MyD88, NF-κB and MAP kinases which are also involved in signaling from other IL-1 family receptors and Toll-like receptors [3]. In vivo administration of IL-33 in mice induces the secretion of Th2-associated cytokines like IL-4, IL-5 and IL-13 thereby promoting type 2 immune responses against helminths and allergens [3].

IL-33 activates eosinophils and induces more than 500 genes including IL-4 and IL-13 [4–6]. IL-33 promotes eosinophil differentiation [7] and tissue eosinophilia after helminth infection [8,9]. IL-33 further induces eosinophil activation and survival [10,11]. In addition to initiating protective immunity against helminths, IL-33 has been shown to regulate fat and glucose metabolism [12]. Mice impaired in IL-33 signaling due to targeted mutation of the IL-33 receptor subunit ST2 show diminished eosinophil counts in adipose tissue [13]. A similar effect was observed by injection of a ST2 blocking antibody into mice [14].

It has been shown that eosinophils constitutively express mRNAs of the Th2-associated cytokines IL-4 and IL-13 [15]. Eosinophil-derived IL-4/IL-13 appears to be important for maintenance of alternatively activated macrophages involved in glucose homeostasis in adipose tissues [16]. Furthermore, it has been demonstrated that eosinophil-derived IL-4/IL-13 stimulate local proliferation of fibro/adipocyte progenitor cells to induce muscle repair [17]. Another study revealed that eosinophils are recruited to liver lesions where they release IL-4/IL-13 to induce hepatocyte proliferation, an important step in liver regeneration [18]. Given these manifold functions of eosinophils in immunity against helminths, allergy, tissue regeneration and metabolism, specific interventions to modulate survival and IL-4/IL-13 release from eosinophils could be a promising therapeutic approach. At present, the mechanisms of IL-33-induced eosinophil survival and IL-4/IL-13 secretion remain poorly understood.

To investigate this issue we performed in vitro experiments with bone marrow derived eosinophils from wild-type or genetically modified mouse strains. We observed that IL-33 acts directly on eosinophils and protects them from spontaneous apoptosis upon growth factor deprivation. This effect was dependent on MyD88 signaling and was also observed with LPS stimulation. Autocrine production of GM-CSF was required for IL-33-mediated expression of Bcl-x_L_ and inhibition of apoptosis. IL-33 and LPS also synergistically induced p38 kinase-dependent secretion of IL-4 and IL-13 from eosinophils. Unexpectedly, cross-linking of the sialic acid binding receptor Siglec-F enhanced the IL-33-induced cytokine release. Finally, co-culture experiments with purified eosinophils and macrophages revealed that IL-33-induced differentiation of alternatively activated macrophages was dependent on eosinophil-derived IL-4/IL-13 and enhanced in the presence of GM-CSF.

## Materials and Methods

### Mice

Wild-type BALB/c and C57BL/6 mice were purchased from Charles River, Sulzfeld, Germany. MyD88^-/-^ [19], ST2^-/-^ [20] and IL-4/IL-13^-/-^[21] mice have been described. Mice were kept under specific pathogen free conditions and both males and females were used at 8–12 weeks of age for experiments.

### Ethics statement

All experiments were performed in accordance with German animal protection law and European Union guidelines 86/809 and were approved by the Federal Government of Lower Franconia.

### Eosinophil culture

Eosinophils were generated from bone marrow as described before [22]. In brief, bone marrow was flushed out from femur and tibia. Single cell suspension was generated and erythrocytes removed by hypotonic lysis. Bone marrow cells were placed in BM-medium (RPMI 1640 (Pan Biotech, Aidenbach, Germany) containing 20% fetal bovine serum (FCS), 55 μM β-mercaptoethanol, 10 mM non-essential amino acids, 1 mM sodium pyruvate, 100 IU/ml penicillin, 100 μg/ml streptomycin, 2 mM glutamine (all from Life technologies, Darmstadt, Germany) and 25 mM Hepes buffer). Cells were stimulated with 100 ng/ml SCF and 100 ng/ml FLT3L (both from Peprotech, Rocky Hill, NJ) for 4 days. From day 4 on cells were kept in BM-medium supplemented with 10 ng/ml recombinant murine IL-5 (R&D Systems, Minneapolis, MN). At day 14 the cultures contained >97% bone marrow-derived eosinophils (BMDE) indicated by high side scatter profile and expression of Siglec-F (S1 Fig).

### BMDE stimulation

BMDE were harvested and washed once in BM-medium. 2x10^5^ cells per well were seeded in 96 well plates. After resting for 2 hours cells were stimulated with indicated cocktails of IL-5, IL-33 (both from R&D Systems) and GM-CSF (Immunotools, Friesoythe, Germany), all at 10 ng/ml. Supernatants were collected and stored at -80°C. In some experiments purified α-Siglec-F (clone E50.2440, BD Bioscience, San Jose, CA) was used at 5 μg/ml to cross-link Siglec-F and ratIgG2a isotype control (clone 2A3, BioXcell, West Lebanon, NH) served as control. To block GM-CSF signaling cells were incubated with α-GM-CSF receptor alpha chain antibody (clone 698423, R&D Systems) at 50 μg/ml for 2 hours before stimulation with cytokines. To block p38 kinase activity eosinophils were pre-incubated with the p38 inhibitor SB203580 in DMSO (Sigma-Aldrich, St.Louis, MO) for 2 hours at 5 μM before cytokines were added. Controls were treated with the same amount of DMSO. LPS (Sigma-Aldrich) was used at 10 μg/ml or at indicated concentrations.

### Generation of bone marrow derived macrophages (BMDM)

Bone marrow derived macrophages were generated as previously described with some modifications [23]. In brief, single cell suspensions from bone marrow were prepared as described for generation of BMDE. Bone marrow cells were resuspended in BM-medium containing 10% supernatant of M-CSF producing fibroblast cell line L929. After 7 days of culture adherent cells were harvested by Accutase (Sigma-Aldrich, St. Louis, MO) treatment. Purity of BMDM (F4/80^+^CD11b^+^) was >95% as determined by flow cytometry.

### BMDE:BMDM co-culture

2x10^5^ BMDM were seeded in each well of a 24 well plate. After 4 hours 1x10^6^ BMDE were added and stimulated with the indicated cytokine cocktails for 24 hours. In separate cultures supernatants from stimulated BMDE were added to BMDM. Cells were harvested and RNA was prepared using the RNeasy Mini Kit (Qiagen, Hilden, Germany) according to manufacturer’s protocol.

### ELISA

IL-13 concentrations were measured with an ELISA Development Kit (Peprotech). IL-4 ELISA were performed with α -IL-4 (clone 11B11, BioXcell) as coating antibody and biotinylated α-IL-4 (clone BVD6-24G2, Southern Biotech, Birmingham, AL) as detection Ab. Alkaline phosphatase-coupled streptavidin and para-Nitrophenylphosphat substrate (both from Southern Biotec) were used for detection. GM-CSF concentrations were measured with a commercial ELISA kit (R&D Systems). ELISAs were measured with a Multiskan FC multiplate photometer (ThermoScientific, Waltham, MA).

### Flow cytometry

BMDE or BMDM were harvested and washed once in FACS buffer (PBS/2% FCS/1 mg/ml sodium azide), incubated with anti-CD16/CD32 blocking mAb (2.4G2, BioXcell) for 5 min at room temperature, and stained with diluted mAb mixtures. The following mAbs were used: PE- or Alexa647-coupled anti-mouse-Siglec-F (clone E50-2440, BD Biosciences, San Jose, CA) to determine BMDE purity and Alexa488-coupled anti-mouse-CD11b and APC/Cy7-coupled anti-mouse-F4/80 to determine BMDM purity. Apoptotic cells were stained with APC-Annexin V (Immunotools) in binding buffer (10 mM HEPES, 140 mM NaCl, 2.5 mM CaCl_2_) according to manufacturer’s protocol. Propidium iodide (eBioscience, San Diego, CA) was added to identify dead cells. Samples were acquired on a FACS Canto II instrument (BD Immunocytometry Systems, San Jose, CA) and analyzed by FlowJo software (Tree Star, Ashland, OR).

### Quantitative real-time PCR

RNA was prepared using the RNeasy Mini Kit (Qiagen) according to manufacturer’s protocol. RNA was reversely transcribed into cDNA using the High Capacity cDNA Reverse Transcription Kit (Applied Biosystems, Foster City, CA). Real-time PCR was performed with a CFX connect machine (Biorad, Hercules, CA). Cycling conditions were as follows: 95°C for 30 sec, 50°C (PBGD, Arg-1, Relm-α) or 58°C (Bcl-x_L_, HPRT1) for 45 sec, 72°C for 45 sec. The following primers were used: PBGD for: TGG TTG TTC ACT CCC TGA AGG; PBGD rev: AAA GAC AAC AGC ATC ACA AGG GT; Arg-1 for: GTA TGA CGT GAG AGA CCA CG; Arg-1 rev: CTC GCA AGC CAA TGT ACA CG; Relm-α for: CCA TAG AGA GAT TAT CGT GGA; Relm-α rev: TGG TCG AGT CAA CGA GTA AG; Bcl-x_L_ for: GAC AAG GAG ATG CAG GTA TTG G; Bcl-x_L_ rev: TCC CGT AGA GAT CCA CAA AAG T; HPRT1 for: GTT GGA TAC AGG CCA GAC TTT GTT; HPRT1 rev: GAG GGT AGG CTG GCC TAT AGG CT.

### Statistics

Student*′*s t-test was performed with SigmaPlot (Systat Software Inc., San Jose, CA, USA) software and *P*-values of less than 0.05 were considered statistically significant.

## Results

### IL-33 mediates eosinophil survival in a MyD88- and ST2-dependent manner

**Fig 1:**
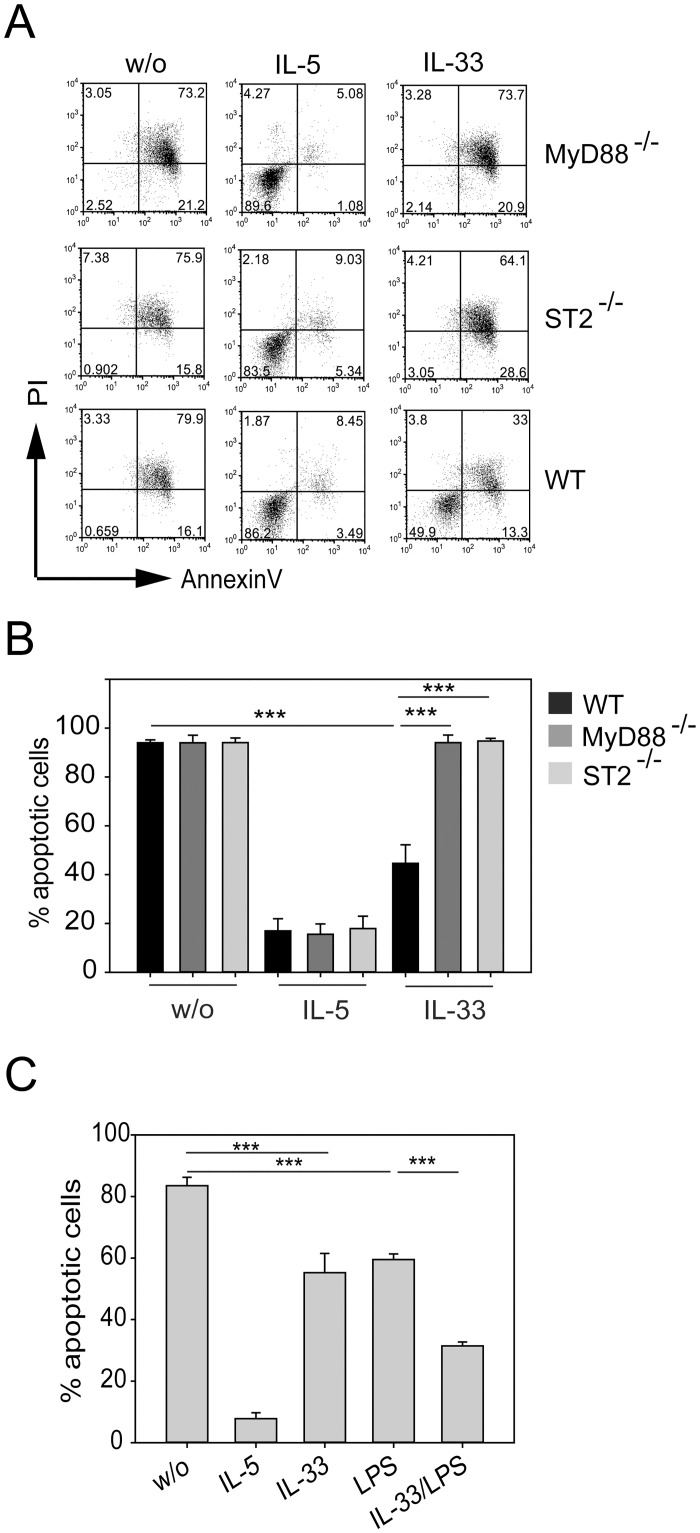
IL-33 mediates eosinophil survival that depends on ST2 and MyD88. BMDE from wild-type (WT), ST2-deficient (ST2^-/-^) and MyD88-deficient (MyD88^-/-^) mice were cultured in presence of 10 ng/ml IL-5 or IL-33 or were left untreated for 72 hours. Cells were harvested and analyzed by flow cytometry to detect apoptotic cells. (A) Dot plots show representative AnnexinV/PI stainings for each experimental group. (B) Bars show the mean + SEM of apoptotic cells from indicated mice. Data are pooled from two independent experiments (n = 5–6; *** p<0.001). (C) BMDE from wild-type mice were stimulated with the indicated cytokines or 10 μg/ml LPS. Control samples were left untreated (w/o). After 72 hours cells were analyzed by flow cytometry to detect Annexin V/PI positive cells. Bars show the mean + SEM of Annexin V^+^ cells pooled from 2–3 independent experiments (n = 8–10; *** p<0.001).

To determine the effect of IL-33 on survival of eosinophils we first generated bone marrow derived eosinophils (BMDE) by culturing bone marrow cells for 10 days in the presence of IL-5 which leads to massive expansion of >97% pure eosinophils. Eosinophils were then cultured in the absence of IL-5 but in the presence of 10 ng/ml IL-33 to investigate whether IL-33 protects from spontaneous apoptosis resulting from IL-5 withdrawal. BMDE from wild-type C57BL/6 mice were compared to BMDE from ST2- or MyD88-deficient mice. The rate of cell death was analyzed at 72 hours after IL-5 withdrawal by AnnexinV/PI staining and flow cytometry. In the absence of IL-5 and IL-33 over 90% of eosinophils became Annexin V^+^ indicative of ongoing cell death while most eosinophils survived in the presence of IL-5 independently of ST2 or MyD88. IL-33 alone could rescue about 50% of eosinophils from cell death but this effect was only observed with BMDE from wild-type mice (Fig 1A and 1B).

Since MyD88 is also involved in signaling from other IL-1 receptor family members and TLRs we further investigated whether LPS shows a similar effect. Indeed, efficient protection from apoptosis could be observed with 10 μg/ml LPS and the combination of IL-33 and LPS revealed an additive effect on the survival of eosinophils (Fig 1C). This indicated that IL-33- or LPS-signaling through MyD88 plays an important role for protection of eosinophils against spontaneous apoptosis.

### IL-33-induced autocrine GM-CSF production mediates eosinophil survival by a Bcl-x_L_- dependent mechanism

**Fig 2:**
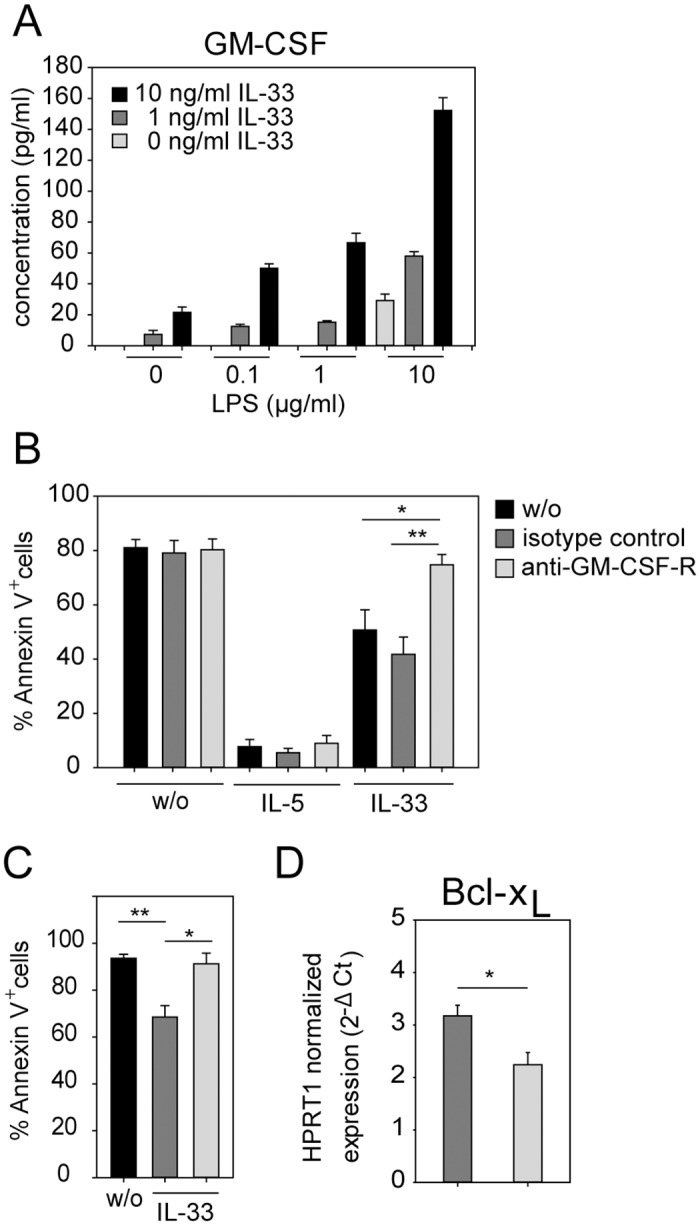
IL-33 mediates eosinophil survival via autocrine GM-CSF production. (A) BMDE were cultured in medium containing 10 ng/ml IL-5 and indicated concentrations of IL-33 and LPS for 24 hours. Supernatants were analyzed by GM-CSF-specific ELISA. Bar graph shows the mean + SD from one of two independent experiments (n = 4;). (B) BMDE were stimulated with the indicated cytokines in the absence (black bars) or presence of a isotype control antibody (dark grey bars) or GM-CSF receptor blocking antibody (light grey bars). After 72 hours cells were analyzed by flow cytometry to detect Annexin V^+^ cells. Bars show the mean + SEM of apoptotic cells from three independent experiments (n = 6; ** p<0.01). (C) Sort-purified BMDE (100% purity, see S1 Fig) were left untreated (black bar) or stimulated with IL-33 in the presence of isotype control antibody (dark grey bar) or anti-GM-CSF-R antibody (light grey bar). After 72 hours cells were analyzed by flow cytometry to detect Annexin V^+^ cells. Bars show the mean + SEM of apoptotic cells from two experiments (n = 4; * p<0.05; ** p<0.01). (D) BMDE were treated as described above and harvested after 24 hours to analyze Bcl-x_L_ transcripts by quantitative RT-PCR. Bar graphs show mean + SEM Bcl-x_L_ mRNA expression levels normalized to HPRT1 mRNA levels with data pooled from two independent experiments (n = 6; * p<0.05).

We could previously show that GM-CSF is a potent inhibitor of spontaneous apoptosis in eosinophils [24]. Here we observed that IL-33 but not IL-5 induced the secretion of GM-CSF from eosinophils and LPS stimulation further enhanced this effect (Fig 2A). This led us to hypothesize that autocrine GM-CSF was required for IL-33-mediated protection from apoptosis. To test this hypothesis, BMDE were treated with IL-33 for 72 hours in the presence of a GM-CSF receptor blocking antibody. We observed that treatment with a GM-CSF receptor blocking antibody completely abolished the anti-apoptotic effect of IL-33 (Fig 2B). In contrast, blocking the GM-CSF signaling pathway had no impact on IL-5-mediated eosinophil survival. To exclude the possibility that the few (<3%) contaminating non-eosinophil cells in the culture are the critical source of GM-CSF we sort-purified eosinophils to 100% purity (S1 Fig) and obtained the same results as with the non-sort purified cultures (Fig 2C). We and others have shown that GM-CSF signaling increases the expression level of the anti-apoptotic protein Bcl-x_L_ in eosinophils [24,25]. As expected, Bcl-x_L_ mRNA levels were significantly reduced when the GM-CSF receptor was blocked during IL-33 stimulation (Fig 2D). We conclude that IL-33 does not directly promote eosinophil survival but rather requires autocrine GM-CSF signaling for this effect.

### GM-CSF and Siglec-F signaling enhance the IL-33-induced release of IL-4 and IL-13

**Fig 3:**
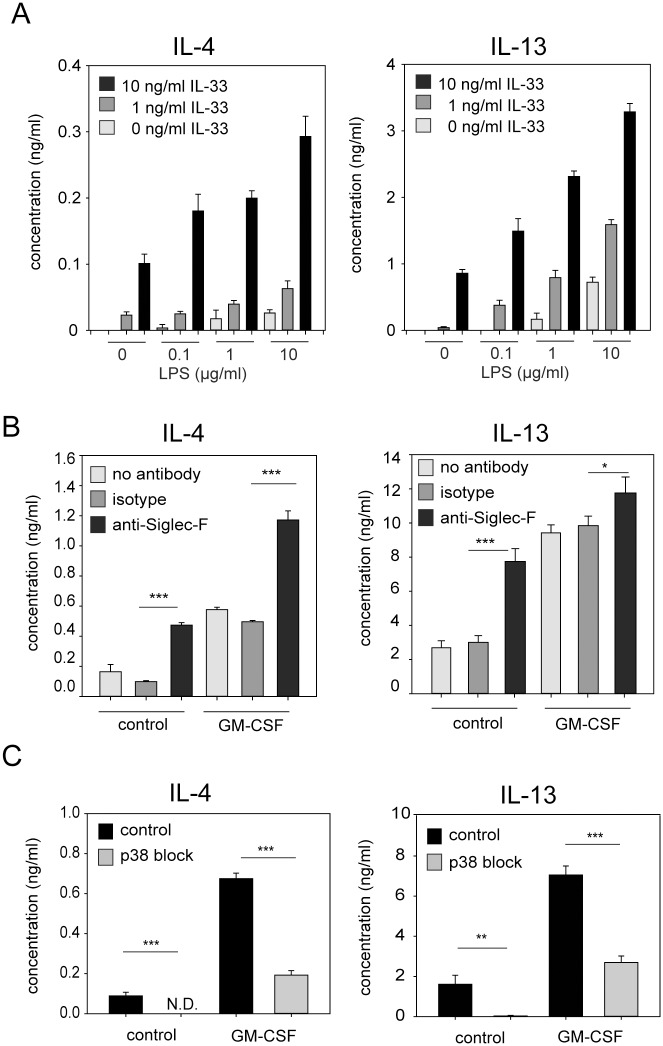
GM-CSF, LPS and Siglec-F signaling enhance IL-33-induced secretion of IL-4 and IL-13 from eosinophils. (A) BMDE were cultured with IL-5 and indicated concentrations of IL-33 and LPS. (B) BMDE were cultured in 10 ng/ml IL-5 and IL-33 in the presence or absence of 10 ng/ml GM-CSF. In addition, anti-Siglec-F, isotype control antibody or no antibodies were added to the culture. (C) BMDE were cultured in 10 ng/ml IL-5 and IL-33. In addition, the p38 kinase was blocked with 5 μM SB203580 and DMSO was used as solvent control. All supernatants were analyzed at 24 hours after onset of culture by IL-4 and IL-13 specific ELISAs. Bars show the mean + SD from one of two independent experiments with similar results (A; n = 4) the mean + SD from one of three independent experiments (B; n = 4) and mean + SEM pooled from two independent experiments (C; n = 8) *p<0.05; **p<0.01; *** p<0.001).

Based on our finding that IL-33 and LPS have an independent and additive effect on eosinophil survival and GM-CSF secretion, we further investigated whether this effect can also be observed for IL-4 and IL-13 secretion from eosinophils. Therefore, BMDE were stimulated with different concentrations of IL-33 and LPS alone or in combination. After 24 hours the concentrations of IL-4 and IL-13 were measured in the supernatant by ELISA. IL-33 and LPS were both potent inducers of IL-4 and IL-13 secretion and the combination of both stimuli revealed an additive effect (Fig 3A).

To further investigate whether IL-33-induced cytokine secretion can be modulated by GM-CSF, we cultured BMDE in IL-5 and IL-33 in the presence or absence of GM-CSF. GM-CSF increased the secretion of IL-4 and IL-13 about 3–4 fold (Fig 3B). Next, we investigated whether cross-linking of the inhibitory receptor Siglec-F dampens IL-33-induced cytokine secretion. Siglec-F is a sialic acid-binding lectin which is highly expressed on eosinophils [26]. Several studies characterized Siglec-F as well as its human orthologue Siglec-8 as apoptosis inducing or inhibitory receptors [27–30]. Surprisingly, anti-Siglec-F stimulated eosinophils also showed 3–4 fold increased cytokine secretion and the combination of GM-CSF and Siglec-F cross-linking revealed an additive effect of both signaling pathways (Fig 3B).

IL-33 can activate the NF-κB and p38 MAP kinase pathway. To address which of these signaling pathways regulates IL-33-induced IL-4/IL-13 release by eosinophils we cultured BMDE in the presence of IL-5 and IL-33 in the presence or absence of the p38 inhibitor SB203580. Inhibition of p38 signaling completely abrogated the IL-33-induced IL-4 and IL-13 release (Fig 3C). This inhibition could be overcome by adding GM-CSF to the culture, although much more IL-4 and IL-13 was produced in the absence of SB203580 (Fig 3C).

### IL-33 activated eosinophils mediate alternative activation of macrophages in vitro

**Fig 4:**
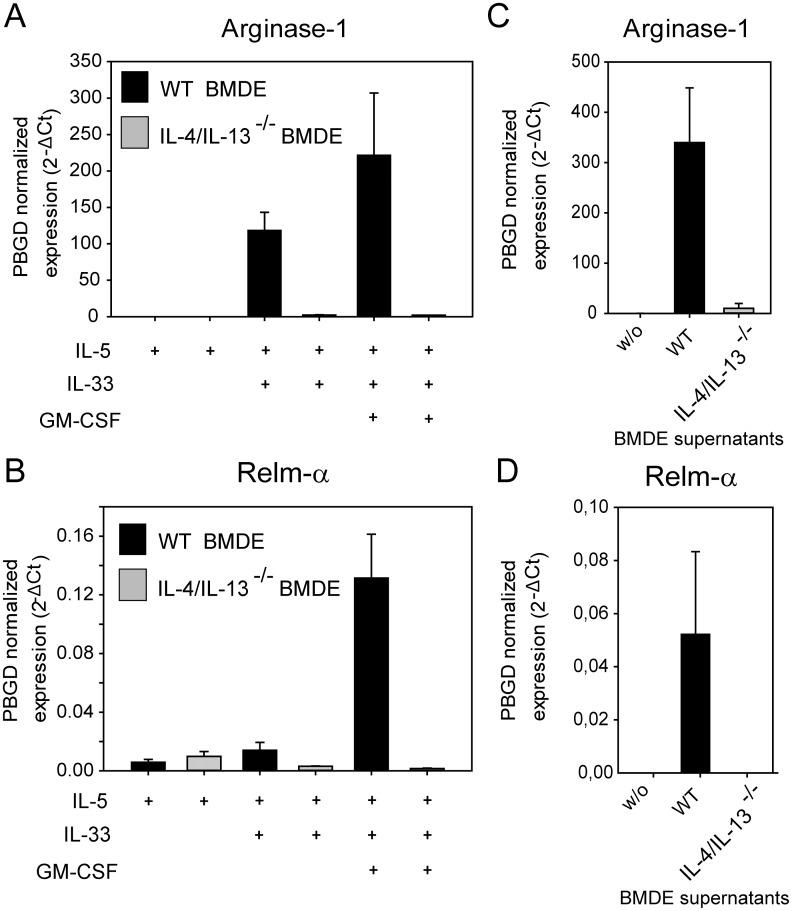
IL-33 activated eosinophils mediate alternative activation of macrophages. (A) and (B) BMDM generated from wild-type BALB/c mice were co-cultured with BMDE from either wild-type (black bars) or IL-4/IL-13-deficient BALB/c mice (grey bars) for 24 hours in the presence of the indicated cytokines. Quantitative RT- PCR was performed to analyze Relm-α and Arginase-1 expression as established markers for alternative activation of macrophages. Data are presented as normalized expression to PBGD. Bars show the mean + SD (n = 3) from one of three independent experiments with similar results. (C) and (D) only supernatants of IL-5+IL-33+GM-CSF stimulated BMDE from WT (black bars) or IL-4/IL-13-deficient mice (grey bars) were added to BMDM for 24 hours before quantitative RT-PCR was performed. Bars show the mean + SD (n = 3) from one experiment.

We described that IL-33-induced secretion of IL-4/IL-13 from eosinophils can be amplified by GM-CSF, LPS and Siglec-F cross-linking. Next we asked if eosinophil-derived IL-4/IL-13 can exert a biologically relevant function. Recent studies implicated eosinophils as source of IL-4/IL-13 to induce or maintain alternatively activated macrophages (AAMs) [16]. Therefore we setup a co-culture system of BMDE with bone marrow derived macrophages (BMDM). BMDM were cultured for 24 hours with eosinophils generated either from wild-type mice or IL-4/IL-13-deficient mice in the presence of IL-5, IL-5+IL-33 or IL-5+IL-33+GM-CSF. We further cultured only BMDM in the presence of supernatants from IL-5+IL-33+GM-CSF stimulated eosinophils. The differentiation of AAMs was determined by RT-PCR for the well-established AAM markers Relm-α (also named Fizz-1) and Arginase-1 (Arg-1). IL-5 alone was not sufficient to promote AAM differentiation (Fig 4A and 4B). The same was observed for co-cultures with IL-5+GM-CSF (S2 Fig). However, the combination of IL-5 and IL-33 induced the expression of Arg-1 but only little Relm-α in the presence of wild-type BMDE (Fig 4A and 4B). The combination of IL-5, IL-33 and GM-CSF caused high-level expression of Arg-1 and Relm-α in the presence of BMDE from wild-type but not IL-4/IL-13-deficient mice (Fig 4A and 4B). The same effect was observed when only supernatants of IL-5+IL-33+GM-CSF stimulated BMDE from wild-type mice was added to BMDM (Fig 4C and 4D). This indicates that IL-33 alone is not sufficient for AAM differentiation when IL-4/IL-13 from other cells (in this case BMDE) is missing which was also observed in a previous report [31]. Furthermore, GM-CSF appears to be a critical factor for efficient secretion of biologically active IL-4/IL-13 from eosinophils.

## Discussion

Eosinophils are mainly known for their protective functions against helminths and pro-inflammatory capacity during allergic responses, but they also play important roles in metabolism and tissue homeostasis. Eosinophils rapidly die by spontaneous apoptosis upon withdrawal of growth factors like IL-5 by mechanisms that are incompletely understood [32]. Here, we show that this spontaneous apoptosis could be partially inhibited by the alarmin IL-33 which confirms pervious findings where IL-33-mediated protection from apoptosis was demonstrated for human eosinophils [11,33].

It has been shown that LPS prolongs human eosinophil survival via a GM-CSF-dependent pathway [34] although it remained controversial whether eosinophils or other cell types are the critical source of GM-CSF [35]. We show that the combination of IL-33 and LPS elicits an additive protective effect on mouse eosinophils. Therefore, it appears that eosinophil survival is promoted by extrinsic (LPS) and intrinsic (IL-33) danger signals particularly under conditions of low IL-5 concentrations as it may occur in certain anatomical sites where eosinophils get recruited during infection or tissue injury.

In mouse eosinophils, the IL-33-induced survival was found to be increased in the absence of the dual-specificity phosphatase DUSP5 which acts on ERK1/2 and this effect correlated with increased levels of Bcl-x_L_ [10]. However, it remained unclear whether IL-33 promotes survival directly or indirectly by induction of other cytokines which can act in an autocrine manner to prevent apoptosis. We and others have shown that GM-CSF is a potent inhibitor of apoptosis for mouse eosinophils and GM-CSF-mediated eosinophil survival depends on Bcl-x_L_.[24,25]. Our current experiments revealed that IL-33-induced eosinophil survival was indirectly mediated by autocrine GM-CSF production. In the in vivo situation GM-CSF is also produced by many other cell types which can thereby help to keep eosinophils alive. Along this line, it was recently shown that GM-CSF was critical for eosinophil accumulation and activation in a mouse model of colitis, although the requirement for direct recognition of GM-CSF by eosinophils was not investigated in this study [36].

We further confirmed previous studies demonstrating that IL-33 induces the secretion of IL-4 and IL-13 from eosinophils by a p38 kinase-dependent pathway [5,7]. In addition, we found that GM-CSF markedly increased the IL-33-induced secretion of IL-4 and IL-13 from eosinophils but had no effect in the absence of IL-33. Others have shown that IL-33-induced secretion of IL-13 from mast cells requires IL-3, a cytokine closely related to GM-CSF [37]. It would be interesting to investigate in future studies how the signaling pathways downstream of the IL-33 and GM-CSF receptors converge to promote efficient cytokine secretion in eosinophils. STAT5 could play an important role in this process since IL-5 and GM-CSF are potent STAT5 activators and STAT5-deficient eosinophils fail to acquire IL-4-producing capacity [38].

Eosinophils in the thymus, small intestine and adipose tissue express higher levels of Siglec-F than their companions in the circulation [16,39,40]. We have previously shown that eosinophils recruited to the lungs of *Nippostrongylus brasiliensis* infected mice show higher levels of Siglec-F compared to eosinophils in naïve mice [41]. In our hands Siglec-F^hi^ eosinophils showed a better survival in vitro as compared to Siglec-F^lo^ eosinophils [41]. However, it was also reported that Siglec-F signaling causes apoptosis in eosinophils [29], although others found that the pro-apoptotic effect of Siglec-F seems to depend on the experimental model [42]. Here we show that Siglec-F cross-linking enhanced IL-33-induced cytokine secretion. To our knowledge, this is the first report of an activating role for Siglec-F. This is surprising since Siglec-F contains a putative immunoreceptor tyrosine-based inhibitory motif (ITIM) in the cytoplasmic tail. Further, Siglec-F does not contain arginine or lysin in the transmembrane domain which would be required for binding to the activating adaptor protein DAP12 as it is known from other activating Siglecs [43]. However, Siglec-F has another cytoplasmic tyrosine-containing motif (SVYTEI) also found in human Siglec-5, -6, -8, -9 and -12. It remains to be determined whether this motif is responsible for the activating property of Siglec-F. We propose a dual role for Siglec-F as an activating and inhibitory receptor similar to what has been described for PIR-B [44].

In conclusion, we found that IL-33-induced survival of eosinophils requires signaling through the GM-CSF receptor and autocrine secretion of GM-CSF is sufficient for this effect. Furthermore, we observed that GM-CSF enhances the secretion of IL-4 and IL-13 from IL-33-stimulated eosinophils which also leads to augmented differentiation of AAMs in co-culture experiments. The observation that Siglec-F signaling promotes cytokine secretion from IL-33-activated eosinophils warrants further investigation to reveal how signaling down-stream of Siglec-F integrates with signaling from the receptors for IL-33 and GM-CSF. A better understanding of mechanisms that regulate eosinophil survival and cytokine secretion are urgently needed to identify potential targets for therapeutic interventions against eosinophil-associated diseases.

## Supporting Information

S1 Fig Analysis of eosinophil purity before and after cell sorting. BMDE cultures were analyzed on day 14 after setup of culture before (left) or after (right) fluorescence-activated cell sorting on a high-speed sorter (S3 sorter from Bio-Rad). Cultures were stained with anti-Siglec-F to detect eosinophils (Siglec-F+SSChi).
(PDF)

S2 Fig IL-5 and GM-CSF are not sufficient for AAM differentiation. BMDE from wild-type (WT) or IL-4/IL-13^-/-^ mice were co-cultured with BMDM from WT mice in the presence of IL-5, IL-5+GM-CSF or IL-5+GM-CSF+IL-33 and expression of Arg-1 and Relm-α was analyzed by quantitative RT-PCR.
(PDF)
