# Supplementary figures and images for: IL-33-Induced Cytokine Secretion and Survival of Mouse Eosinophils Is Promoted by Autocrine GM-CSF
Source: PLoS One. 2016 Sep 30;11(9):e0163751. doi: 10.1371/journal.pone.0163751 (PMC5045177; doi:10.1371/journal.pone.0163751)

Figure S1

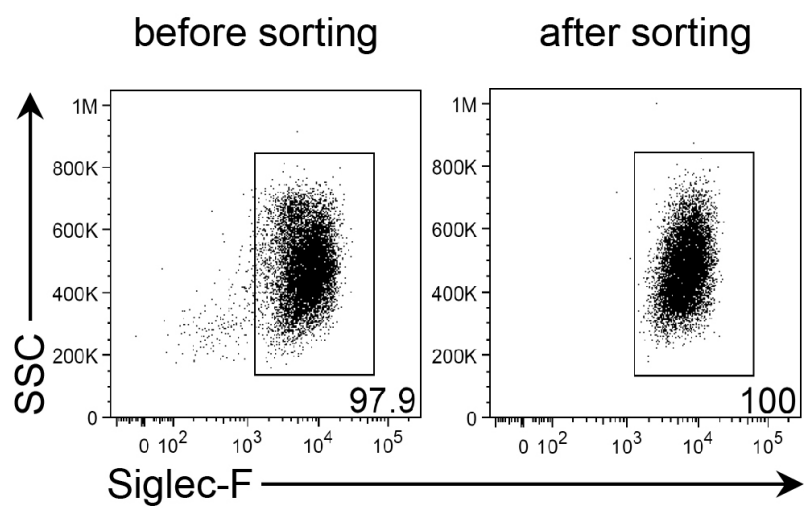

Supplement: S1 Fig — BMDE cultures were analyzed on day 14 after setup of culture before (left) or after (right) fluorescence-activated cell sorting on a high-speed sorter (S3 sorter from Bio-Rad). Cultures were stained with anti-Siglec-F to detect eosinophils (Siglec-F+SSChi). (PDF) [file pone.0163751.s001.pdf]

Figure S2

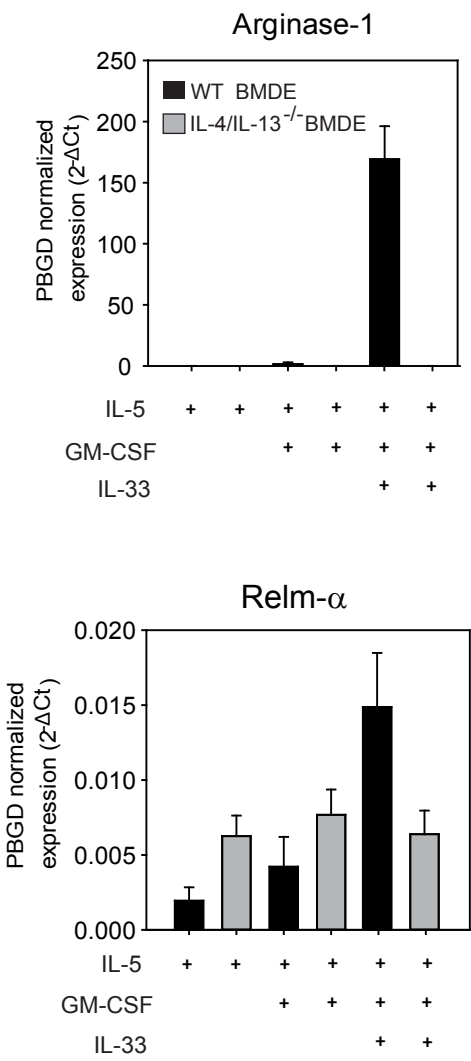

Supplement: S2 Fig — BMDE from wild-type (WT) or IL-4/IL-13-/- mice were co-cultured with BMDM from WT mice in the presence of IL-5, IL-5+GM-CSF or IL-5+GM-CSF+IL-33 and expression of Arg-1 and Relm-α was analyzed by quantitative RT-PCR. (PDF) [file pone.0163751.s002.pdf]
